# Brachial-to-ankle pulse wave velocity as an independent prognostic factor for ovulatory response to clomiphene citrate in women with polycystic ovary syndrome

**DOI:** 10.1186/1757-2215-7-74

**Published:** 2014-07-10

**Authors:** Toshifumi Takahashi, Hideki Igarashi, Shuichiro Hara, Mitsuyoshi Amita, Koki Matsuo, Ayumi Hasegawa, Hirohisa Kurachi

**Affiliations:** 1 Department of Obstetrics and Gynecology, Yamagata University Faculty of Medicine, Yamagata 990-9585, Japan

**Keywords:** Polycystic ovary syndrome, Clomiphene citrate, Brachial-to-ankle pulse wave velocity, Waist-to-hip ratio

## Abstract

**Background:**

Polycystic ovary syndrome (PCOS) has a risk for cardiovascular disease. Increased arterial stiffness has been observed in women with PCOS. The purpose of the present study was to investigate whether the brachial-to-ankle pulse wave velocity (baPWV) is a prognostic factor for ovulatory response to clomiphene citrate (CC) in women with PCOS.

**Methods:**

This study was a retrospective cohort study of 62 women with PCOS conducted from January 2009 to December 2012 at the university hospital, Yamagata, Japan. We analyzed 62 infertile PCOS patients who received CC. Ovulation was induced by 100 mg CC for 5 days. CC non-responder was defined as failure to ovulate for at least 2 consecutive CC-treatment cycles. The endocrine, metabolic, and cardiovascular parameters between CC responder (38 patients) and non-responder (24 patients) groups were analyzed.

**Results:**

In univariate analysis, waist-to-hip ratio, level of free testosterone, percentages of patients with dyslipidemia, impaired glucose tolerance, and diabetes mellitus, blood glucose and insulin levels at 60 min and 120 min, the area under the curve of glucose and insulin after 75-g oral glucose intolerance test, and baPWV were significantly higher in CC non-responders compared with responders. In multivariate logistic regression analysis, both waist-to-hip ratio (odds ratio, 1.77; 95% confidence interval, 2.2–14.1; P = 0.04) and baPWV (odds ratio, 1.71; 95% confidence interval, 1.1–2.8; P = 0.03) were independent predictors of ovulation induction by CC in PCOS patients. The predictive values of waist-to-hip ratio and baPWV for the CC resistance in PCOS patients were determined by the receiver operating characteristic curves. The area under the curves for waist-to-hip ratio and baPWV were 0.76 and 0.77, respectively. Setting the threshold at 0.83 for waist-to-hip ratio offered the best compromise between specificity (0.65) and sensitivity (0.84), while the setting the threshold at 1,182 cm/s for baPWV offered the best compromise between specificity (0.80) and sensitivity (0.71).

**Conclusions:**

Both metabolic and cardiovascular parameters were predictive for CC resistance in PCOS patients. The measurement of baPWV may be a useful tool to predict ovulation in PCOS patients who receive CC.

## Background

Polycystic ovary syndrome (PCOS) is a common ovulatory disorder in young women, which affects 5–10% of the population and results in infertility due to anovulation [1]. Clomiphene citrate (CC) is a first-line strategy to induce ovulation in women with PCOS. Although CC will induce ovulation in 60–80% of PCOS patients, the remainder are CC resistant [2,3]. CC-resistant women are recommended for other treatments, such as gonadotropin treatment or laparoscopic ovarian drilling to induce ovulation. Although gonadotropin therapy achieves higher ovulation and pregnancy rates, the treatment is associated with serious complications, such as ovarian hyperstimulation syndrome and multiple pregnancies [4,5]. Laparoscopic ovarian drilling is invasive and expensive [5,6].

Although the pathogenesis of PCOS is still unclear, the role of insulin resistance in the pathophysiology of PCOS has been established. Insulin resistance with compensatory hyperinsulinemia is common in women with PCOS [7]. Hyperinsulinemia directly contributes to an increase in androgen biosynthesis in the ovary [8] and a decrease in the level of hepatic sex hormone-binding globulin, resulting in an elevated free androgen level [9]. Hyperandrogenism within an ovary is believed to harm follicle development in PCOS. On the basis of these considerations, insulin-sensitizing drugs, such as metformin, pioglitazone, and rosiglitazone, have been used alone or in combination with CC in CC-resistant PCOS patients to induce ovulation [5].

The mechanisms of CC resistance in women with PCOS also remain unknown. Levels of testosterone [10] and blood glucose and blood glucose × immunoreactive insulin (IRI) at 120 min after oral glucose tolerance test have been reported as predictive markers in CC-resistant PCOS [11]. Because PCOS is associated with risk for developing type 2 diabetes mellitus [12], these reports support the idea that metabolic disorders could be involved in the mechanisms of CC-resistant PCOS.

Women with PCOS also are at risk for developing cardiovascular disease [13-16]. Increased arterial stiffness has been observed in women with PCOS compared to controls with normal menstrual cycles and normal androgen levels [17,18]. Brachial-to-ankle pulse wave velocity (baPWV) is a surrogate marker for arterial stiffness [19]. However, no reports concerning the association of cardiovascular parameters, such as baPWV, with ovulatory response to CC in women with PCOS have been published. The purpose of the present study was to investigate whether the baPWV is a prognostic factor for ovulatory response to CC in women with PCOS.

## Methods

### Subjects

This study was a retrospective cohort observational study. A total of 62 infertile patients diagnosed with PCOS from January 2009 to December 2012 in our hospital were recruited into the study. The ethics committee at Yamagata University Faculty of Medicine approved the study protocol. We previously reported that this cohort of seven women with PCOS who were CC-resistant successfully ovulated by co-treatment with bezafibrate and CC [20]. A written informed consent was obtained from all participants before entry into the study.

PCOS was diagnosed by the presence of oligomenorrhea or amenorrhea, polycystic ovaries on ultrasonography, and hyperandrogenemia or LH hypersecretion (elevated LH level and LH/FSH ratio) according to the 2007 criteria of the Japanese Society of Obstetrics and Gynecology (JSOG) [21]. Pelvic transvaginal ultrasound was performed using a 5 to 7.5-MHz probe in oligo-/amenorrheic women at random or days 3–5 after a spontaneous or a progestin-induced withdrawal bleeding. In the JSOG PCOS diagnostic criteria, a diagnosis of PCO was made if there were 10 or more follicles of 2–9 mm in diameter [21]. Because the prevalence of Japanese PCOS women with a sign of hyperandrogenism is very low [22], the clinical hyperandrogenism, such as hirsutism, is not included in the JSOG PCOS diagnostic criteria. Hyperandrogenemia was defined as an increase in the level of total testosterone, free testosterone or androstenedione concentration in the JSOG PCOS diagnostic criteria [21]. In the present study, we used the concentration of free testosterone over 1.0 pg/ml as hyperandrogenemia. The JSOG PCOS diagnostic criteria includes the Rotterdam 2003 criteria for PCOS [23]. Other etiologies such as congenital adrenal hyperplasia, androgen secreting tumor, or Cushing’s syndrome were excluded [21,23]. Inclusion criteria included normal semen analysis according to World Health Organization criteria and normal hysterosalpingography within the preceding 6 months. Exclusion criteria included the presence of any infertility factors other than PCOS and the use of any medications, such as insulin sensitizers, lipid-lowering drugs, or anti-hypertensive drugs. Patients who were already diagnosed with impaired glucose tolerance or diabetes mellitus and smokers were also excluded from the study.

All patients were received CC (Shionogi Co. Ltd., Tokyo Japan) at 100 mg daily for 5 days from day 3–5 after a spontaneous or a progestin-induced withdrawal bleeding [20,24]. The patients were followed up with transvaginal ultrasound to record follicular growth from day 10–12 of the cycle. When a follicle of at least 18 mm was found, 5,000 IU of human chorionic gonadotropin (hCG, Mochida Co. Ltd., Tokyo Japan) was intramuscularly injected [25]. Artificial insemination with the husband’s semen or natural intercourse was performed after the hCG injection. Serum progesterone was measured 7 days after hCG administration. Ovulation was determined as the vanishing of a follicle on transvaginal ultrasound, a rise in basal body temperature, and serum progesterone level >10 ng/ml at day 7 after hCG administration [20]. If no follicular growth was observed at day 21 of the menstrual cycle, we discontinued the measurement of follicle growth and recorded no ovarian response to CC. CC response was defined as confirmed ovulation in at least one cycle of CC administration [11]. CC non-response was defined as failure to demonstrate an ovarian response, and no ovulation for at least two consecutive cycles [20,26].

### Hormonal assays

In the first cycle of CC treatment, all hormone measurements were performed on day 3–5 after a spontaneous or a progestin-induced withdrawal bleeding. Hormone concentrations were quantified using commercially available immunoassay kits. LH, FSH, PRL, and total testosterone were measured using an electrochemiluminescence immunoassay (ECLusys reagent LH, FSH, prolactin, testosterone II kit; Roche Diagnostics, Inc., Tokyo, Japan). Estradiol and progesterone levels were measured using a chemiluminescence immunoassay (Architect estradiol and progesterone kit; Abbott Japan, Inc., Tokyo, Japan). Free testosterone and dehydroepiandrosterone sulfate (DHEA-S) were measured by radioimmunoassay (DPC Free Testosterone Kit and DHEA-S kit; Mitsubishi Kagaku Iatron, Inc., Tokyo, Japan). Reliability criteria for total testosterone and free testosterone assays were established. The intra-assay coefficient of variation (CV) was 4.7–5.3% and the detection limit of the assay was 10 ng/dl for total testosterone, whereas the intra-assay CV was 5.7–7.4% and the detection limit of the assay was 0.24 pg/ml for free testosterone. All samples were assayed in duplicate.

### Anthropometric measurements

In the first cycle of CC treatment, anthropometric measurements were performed on day 3–5 after a spontaneous or a progestin-induced withdrawal bleeding. Body height was measured to the nearest 0.5 cm using a stadiometer, and body weight (in light clothing without shoes) to the nearest 0.5 kg on a calibrated balance scale. Each patient’s waist circumference was measured with a soft tape midway between the lowest rib and the iliac crest in the standing position. Hip circumference was measured at the widest part of the gluteal region, and the waist-to-hip ratio was calculated. Body mass index (BMI, kg/m^2^) was calculated as weight in kilograms divided by the square of height in meters. Obesity was defined as BMI ≥ 30 kg/m^2^.

### Measurements of metabolic parameters

In the first cycle of CC treatment, a blood sample was collected by venipuncture on day 3–5 after a spontaneous or a progestin-induced withdrawal bleeding. Serum levels of triglycerides, low-density lipoprotein (LDL) cholesterol, high-density lipoprotein (HDL) cholesterol, and total cholesterol were measured by an enzymatic assay. The diagnostic criteria for dyslipidemia were LDL cholesterol ≥ 140 mg/dl, HDL cholesterol < 40 mg/dl, or triglycerides ≥ 150 mg/dl according to the Japan Atherosclerosis Society’s Guidelines for the Diagnosis and Prevention of Atherosclerotic Cardiovascular Diseases in Japanese [27].

Fasting plasma glucose (FPG) was measured by enzyme assay, and fasting IRI was measured by enzyme immunoassay. Glucose tolerance was determined by a 75-g oral glucose tolerance test (75-g OGTT). The diagnostic criteria for diabetes mellitus were in accordance with the Committee of the Japan Diabetes Society on the Diagnostic Criteria of Diabetes Mellitus [28]. Impaired glucose tolerance was defined as a 2-h postload plasma glucose level ≥ 140 mg/dl in subjects not meeting the criteria for diabetes mellitus [28]. The responses of glucose and insulin to 75-g OGTT were analyzed by calculating the area under the curve (AUC) for glucose and the AUC for insulin using a trapezoidal method. Insulin resistance was determined by homeostasis model assessment of insulin resistance (HOMA-IR) and quantitative insulin-sensitivity check index (QUICKI). The HOMA-IR was calculated using the formula FPG (mg/dl) × fasting IRI (μU/ml)/405, while the QUICKI was calculated as 1/(log fasting IRI [μU/ml] + log FPG [mg/dl]). Patients with HOMA-IR > 2.0 were considered insulin resistant. Fasting venous blood samples were taken on a random day, between 8:30 and 10:30 AM after a 12-h overnight fast, before initiation of ovulation induction with CC.

### Measurements of cardiovascular parameters

In the first cycle of CC treatment, measurements of cardiovascular parameters were performed on day 3–5 after a spontaneous or a progestin-induced withdrawal bleeding. Blood pressure was measured in the supine position on the right arm after a 10-min rest; a standard sphygmomanometer of appropriate cuff size was used, and the first and fifth phases were recorded. Values used in the analysis are the average of 3 readings taken at 5-min intervals. The diagnostic criteria for hypertension were systolic blood pressure ≥ 140 mmHg or diastolic blood pressure ≥ 90 mmHg based on the Japanese Society of Hypertension Guidelines for the Management of Hypertension [29]. Mean blood pressure, which indicates overall peripheral resistance, was calculated as diastolic pressure plus one-third of the pulse pressure. baPWV, which indicates arterial stiffness, was measured using a volume-plethysmographic apparatus (form ABI; Omron Colin, Co., Ltd., Tokyo, Japan). The patient was examined in the supine position after resting for at least 5 min, with electrocardiogram electrodes placed on both wrists, a microphone to detect heart sounds placed on the left edge of the sternum, and cuffs wrapped on both the brachia and ankles. The intra- and interassay CV were < 8% for baPWV.

### Statistical analysis

Because there were no studies in the literature on this subject, we were unable to estimate pretest power. Therefore, post hoc power analysis was performed by G*Power Software (version 3.1.9.2). Data were presented as mean ± SD if a normal distribution was expected; otherwise, median and range were used. In univariate analysis, differences in nominal variables between the groups were compared with the χ^2^ test, unless the expected frequency was < 5, in which case, the Fisher’s exact probability test was used. Differences in continuous variables were analyzed by nonparametric Mann-Whitney *U* test. The differences in continuous variables were compared between groups using the Student’s *t* test. Multivariate logistic regression analysis was applied to evaluate the predictors for ovulatory response to CC in PCOS patients. *P* > 0.10 was used as a cutoff level to eliminate non-significant predictors from the prognostic model. The area under the receiver operating characteristic curve was used to assess the discriminative ability of the logistic models. Statistical analysis was performed with R software (The R Foundation for Statistical Computing, version 2.13.0). Significance was defined as *P* < 0.05.

## Results

Thirty-eight PCOS patients (61%) were CC responders, and 24 (39%) were non-responders. Table 1 summarizes the clinical characteristics and endocrine parameters of the CC responder and non-responder groups. Age, BMI, the percentages of overweight (25 ≤ BMI < 30) and obese (BMI ≥ 30) patients did not significantly differ between the groups. Waist-to-hip ratio was significantly higher among CC non-responders than CC responders. Although testosterone concentration did not differ between the groups, the level of free testosterone in CC non-responders was significantly higher than that in CC responders.

**Table 1:** Clinical characteristics and endocrine parameters of CC responsive and non-responsive patients with polycystic ovary syndrome

	**CC responders (n = 38)**	**CC non-responders (n = 24)**	***P *** **value**
**Age (y)**	30.5 ± 4.7	31.5 ± 3.7	0.38
**BMI (kg/m**^**2**^**)**	23.7 ± 5.6	25.7 ± 6.4	0.21
**No. of patients with < 18.5 BMI (%)**	4 (11)	3 (13)	0.83
**No. of patients with 18.5 ≤ BMI < 25 (%)**	22 (58)	9 (38)	0.19
**No. of patients with 25 ≤ BMI < 30 (%)**	6 (16)	7 (29)	0.22
**No. of patients with ≥ 30 BMI (%)**	6 (16)	5 (21)	0.74
Gravida*	0 (0–3)	1 (0–4)	0.26
Parity*	0 (0–1)	0 (0–1)	0.82
**Waist (cm)**	78.2 ± 12.4	84.0 ± 12.3	0.11
**Hip (cm)**	95.82 ± 9.1	96.9 ± 8.8	0.67
**Waist-to-hip ratio**	0.81 ± 0.06	0.88 ± 0.07	0.001
**LH (mIU/ml)**	10.7 ± 3.8	12.3 ± 7.3	0.25
**FSH (mIU/ml)**	7.0 ± 1.7	6.6 ± 1.1	0.32
**LH/FSH**	1.6 ± 0.6	1.8 ± 0.9	0.23
**PRL (ng/ml)**	12.1 ± 5.8	12.3 ± 7.3	0.92
**E2 (pg/ml)**	46.93 ± 20.6	50.2 ± 18.2	0.54
**Testosterone (ng/ml)**	78.5 ± 25.8	99.6 ± 33.9	0.11
**Free testosterone (pg/ml)**	0.80 ± 0.34	1.17 ± 0.69	0.006
**DHEA-S (μg/dl)**	2095 ± 683	2284 ± 814	0.50

CC: clomiphene citrate, BMI: body mass index, DHEA-S: dehydroepiandrosterone sulfate.

*Median (range).

Table 2 summarizes the metabolic parameters in both groups. The percentage of CC non-responders with dyslipidemia was significantly higher than that of CC responders. The lipid parameters of triglycerides, LDL cholesterol, HDL cholesterol, and total cholesterol did not significantly differ between the groups. The percentage of patients with impaired glucose tolerance or diabetes mellitus was significantly higher among CC non-responders than CC responders. In assessing the glycemic and insulinemic response to 75-g OGTT, the levels of fasting glucose and IRI did not differ between groups, while the blood glucose and IRI levels at 60 min and 120 min and the AUC of glucose and IRI were significantly higher among CC non-responders than in CC responders. The values of HOMA-IR and QUICKI, indicators for insulin resistance, did not significantly differ between the groups.

**Table 2:** Metabolic parameters in CC responsive and non-responsive patients with polycystic ovary syndrome

	**CC responders (n = 38)**	**CC non-responders (n = 24)**	***P *** **value**
**No. of patients with dyslipidemia (%)**	6 (16)	11 (38)	0.02
**Triglycerides (mg/dl)**	90.4 ± 44.9	117.1 ± 67.6	0.08
**LDL cholesterol (mg/dl)**	112.2 ± 44.6	116.0 ± 32.9	0.73
**HDL cholesterol (mg/dl)**	65.3 ± 13.5	60.1 ± 15.6	0.18
**Total cholesterol (mg/dl)**	192.0 ± 42.0	197.9 ± 31.6	0.57
**No. of patients with IGT or DM (%)**	4 (11)	11 (46)	0.004
**75-g OGTT**			
**Plasma glucose (mg/dl)**			
**Fasting**	88.9 ± 8.4	89.6 ± 7.5	0.76
**60 min**	129.0 ± 36.3	159.9 ± 48.4	0.01
**120 min**	112.1 ± 21.6	129.7 ± 41.9	0.04
**AUC glucose (mg × h/dl)**	936.9 ± 192.6	1080.9 ± 267.3	0.03
**IRI (μU/ml)**			
**Fasting**	9.6 ± 7.3	13.7 ± 9.7	0.09
**60 min**	67.1 ± 34.7	117.6 ± 90.0	0.007
**120 min**	61.2 ± 36.5	110.7 ± 107.5	0.02
**AUC IRI (μU × h/ml)**	473.3 ± 225.9	799.1 ± 609.1	0.009
**HbA1c (%)**	5.4 ± 0.4	5.6 ± 0.4	0.12
**HOMA-IR**	2.3 ± 1.9	3.4 ± 2.9	0.09
**QUICKI**	0.36 ± 0.04	0.34 ± 0.05	0.16

CC: clomiphene citrate, IGT: impaired glucose tolerance, DM: diabetes mellitus, AUC: area under the curve, IRI: immunoreactive insulin, HbA1c: hemoglobin A1c, HOMA-IR: homeostasis model assessment of insulin resistance, QUICKI: quantitative insulin-sensitivity check index.

Table 3 summarizes the cardiovascular parameters in both groups. The levels of systolic, diastolic, and mean blood pressure did not significantly differ, while baPWV was significantly higher in the CC non-responder group than in CC responders.

**Table 3:** Cardiovascular parameters in CC responsive and non-responsive patients with polycystic ovary syndrome

	**CC responders (n = 38)**	**CC non-responders (n = 24)**	***P *** **value**
**No. of patients with hypertension (%)**	6 (16)	3 (13)	1.0
**Systolic BP (mmHg)**	118.5 ± 15.5	121.2 ± 16.1	0.54
**Diastolic BP (mmHg)**	69.3 ± 14.4	71.0 ± 11.8	0.64
**Mean BP (mmHg)**	85.7 ± 13.8	87.8 ± 12.9	0.58
**baPWV (cm/s)**	1113.9 ± 129.9	1249.2 ± 173.4	0.002

CC: clomiphene citrate, baPWV: brachial-to-ankle pulse wave velocity.

**Figure 1:**
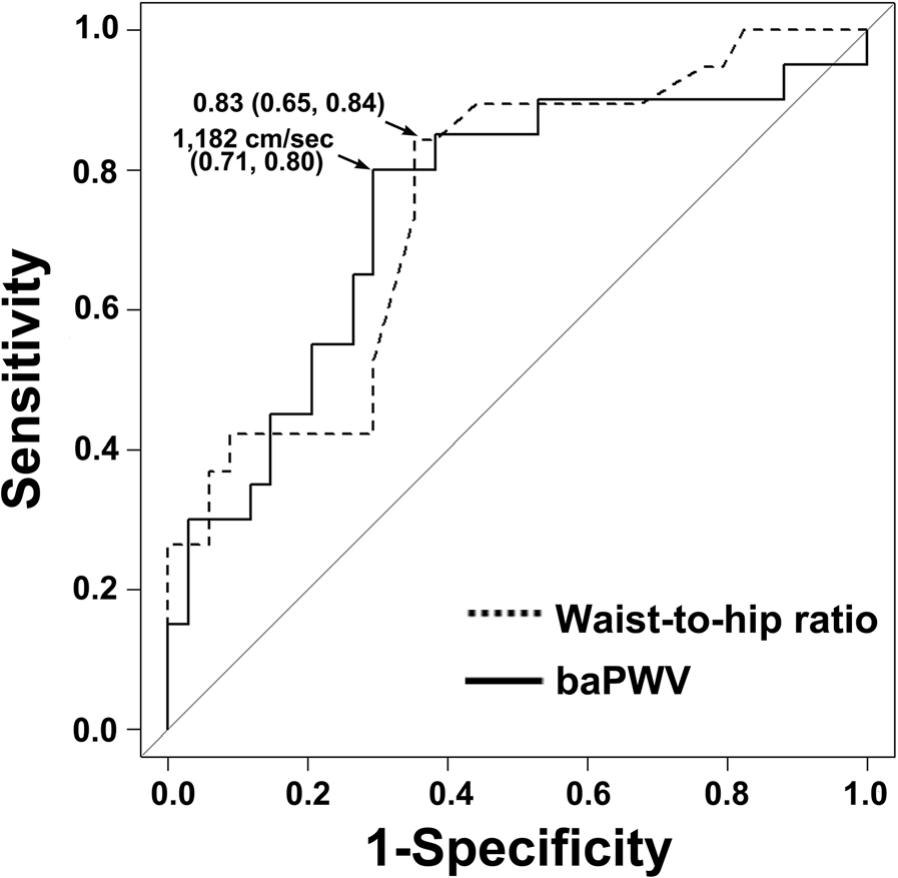
Receiver operating characteristic curve of waist-to-hip ratio and brachial-to-ankle pulse wave velocity (baPWV) to predict clomiphene citrate resistance in patients with polycystic ovary syndrome.

In multivariate logistic regression analysis, both waist-to-hip ratio and baPWV were independent predictors for ovulatory response to CC in the PCOS patients (Table 4). Therefore, we validated the efficacy of measurement of waist-to-hip ratio and baPWV for the prediction of CC resistance in PCOS patients. Figure 1 shows the receiver operating characteristic curves of waist-to-hip ratio and baPWV for the prediction of CC resistance. The AUCs for waist-to-hip ratio and baPWV were 0.76 and 0.77, respectively. Setting the threshold at 0.83 for waist-to-hip ratio offered the best compromise between specificity (0.65) and sensitivity (0.84), while the setting the threshold at 1,182 cm/s for baPWV offered the best compromise between specificity (0.80) and sensitivity (0.71). For patients with waist-to-hip ratio ≥ 0.83, the positive and negative predictive values were 0.72 and 0.79, respectively. For patients with baPWV ≥ 1,182 cm/s, the positive and negative predictive values were 0.69 and 0.81, respectively.

**Table 4:** Independent predictors for ovulatory response to CC in patients with polycystic ovary syndrome

**Independent variables**	**Odds ratio (95% confidence interval)**	***P *** **value**
Waist-to-hip ratio	1.77 (2.2–14.1)	0.04
baPWV	1.71 (1.1–2.8)	0.03

CC: clomiphene citrate, baPWV: brachial-to-ankle pulse wave velocity.

## Discussion

In the present study, we found that both metabolic and cardiovascular parameters were predictive for CC resistance in patients with PCOS. The measurement of waist-to-hip ratio and baPWV may be useful tools to predict the possibility for ovulation in PCOS patients who receive CC.

In the present study, waist-to-hip ratio was an independent predictor for ovulatory response to CC in PCOS patients. The waist-to-hip ratio correlates with visceral adiposity in obese and non-obese women with PCOS [30]. Douchi et al. reported that the ratio of trunk fat to leg fat, which indicates visceral adiposity, is a good predictor for CC resistance in women with PCOS [31], which is consistent with our findings. In their study, the trunk-to-leg fat ratio was measured by dual-energy X-ray absorptiometry. The measurement of waist-to-hip ratio is an easy and more convenient method to predict CC resistance in PCOS. Lord et al. reported that visceral adiposity, but not body weight, BMI, or subcutaneous adiposity, was strongly correlated with insulin resistance in women with PCOS [30]. The increased visceral adiposity induces a decrease in adiponectin, which has an anti-inflammatory action, and an increase in inflammatory cytokines, such as tumor necrosis factor-α, interleukin-6, and free fatty acids, which raise insulin resistance [32]. The insulin resistance of peripheral tissues is thought to be an important etiological factor in PCOS [7]. These results support the hypothesis that increased visceral adiposity may be associated with CC resistance in PCOS women. Furthermore, waist-to-hip ratio is a well-known predictor for cardiovascular disease [33,34].

In the present study, baPWV was also an independent predictor for ovulatory response to CC in patients with PCOS. The baPWV is a surrogate marker for arterial stiffness [19] and a strong independent predictor of cardiovascular mortality in the patients with hypertension, end-stage renal disease, and diabetes [35-37]. An association between PCOS and arterial stiffness has been suggested [17,38-40]. Kelly et al. first reported that the brachial artery, but not aortic artery, PWV is significantly higher in reproductive-aged women with PCOS than in control women with regular menstrual cycles and normal androgen levels [17]. Meyer et al. also reported that central PWV in reproductive-aged women with PCOS is significantly higher than that in control women with regular menstrual cycles and normal androgen levels [39]. Moreover, Sasaki et al. reported that baPWV in reproductive-aged women with PCOS is significantly higher than that in control women with regular menstrual cycles and morphologically normal ovaries [18].

PWV is known to increase with age and hypertension in the general population [41]. Furthermore, several studies have reported that PWV increases in diabetic, dyslipidemia, and metabolic syndrome patients with insulin resistance [42,43]. Both total cholesterol and HOMA-IR, an indicator for insulin resistance, have been reported to correlate with PWV in women with PCOS [39]. Dyslipidemia is a well-known primary risk factor for the development of atherosclerosis and is very common in women with PCOS [44]. Therefore, dyslipidemia observed in reproductive-aged women with PCOS may influence early alteration of arterial structure, which leads to arterial stiffness.

No evidence of an association between PWV and CC resistance in women with PCOS has been previously reported. The precise mechanism for the increase in baPWV among CC non-responders compared to CC responders remains unclear. In the present study, although surrogate markers for insulin resistance, HOMA-IR and QUICKI, did not significantly differ between CC responders and non-responders, the percentage of patients with dyslipidemia and impaired glucose tolerance or diabetes mellitus in the CC non-responder group was higher compared to CC responders. Because PWV increases in patients with dyslipidemia and diabetes mellitus [43,45], these factors may be one of the causes for the increase in baPWV among CC non-responders.

In the present study, free, but not total, testosterone level was also significantly higher in the CC non-responder group compared to CC responders. The free androgen index (FAI), which is obtained as the quotient 100 × total testosterone/SHBG, has been reported as higher in CC-resistant PCOS patients than in CC responders [46]. Moreover, Imani et al. reported that BMI, FAI, menstrual cycle history, and ovarian volume are useful predictors for CC resistance in infertile patients with World Health Organization group-II ovulatory disorders including PCOS [47]. These results indicate that hyperandrogenism, which may impair ovarian follicle development, may be involved in CC resistance in PCOS patients. In reproductive-aged women without PCOS, SHBG levels, but not total or free testosterone levels, were inversely associated with subclinical cardiovascular disease assessed by coronary artery calcified plaques and carotid artery intima-media thickness [48]. Creatsa et al. recently reported that higher serum testosterone and FAI are associated with subclinical atherosclerosis, while serum DHEA-S exhibits a negative association with arterial stiffness in healthy recently menopausal women [49]. These reports suggest that hyperandrogenism may be a link to the increased arterial stiffness observed in CC non-responsive PCOS patients.

In the present study, we could not explore the possible mechanisms involved in the increase in baPWV in CC-resistant PCOS patients. Further investigations are needed to demonstrate to link to the increase in arterial stiffness in CC-resistant PCOS. Because women with PCOS have multiple risk factors for cardiovascular disease (CVD) [14-16], such as impaired glucose tolerance, dyslipidemia, insulin resistance, and metabolic syndrome, the Androgen Excess and Polycystic Ovary Syndrome (AE-PCOS) Society published a consensus statement regarding assessment of CVD risk and CVD in women with PCOS [13].

Our study has certain limitations that should be noted. First, as PCOS was diagnosed according to the 2007 criteria of the JSOG [21], whose criteria matched the Rotterdam 2003 criteria [23], our findings might differ from other PCOS cohorts diagnosed by different criteria, such as National Institutes of Health 1990 and AE-PCOS. Because the prevalence of Japanese PCOS women with a sign of hyperandrogenism is very low compared to Caucasian women [22], the results of this study may not be extrapolated to other populations. Second, definitions vary the dose required to define CC-resistance ranging from 100 mg to 250 mg of CC [20,26,46,50,51]. In the present study, we defined CC non-responder as failure to ovulate with 100 mg of CC for at least 2 consecutive CC-treatment cycles. The CC non-responder in our study may ovulate in response to 150 mg of CC administration. However, the doses in excess of 100 mg per day are not approved by the Ministry of Health, Labour and Welfare of Japan as well as Food and Drug Administration of United States. Therefore, we could not prescribe more than 100 mg per day of CC in this study. Lastly, because of small sample size and retrospective study design, our findings should be interpreted with caution and confirmed by prospective study.

We performed a post hoc power analysis for the waist-to-hip ratio, baPWV, blood glucose and IRI levels at 60 min, and AUC of glucose and IRI with a two-sided level of significance of 0.05 and found a power of 0.98, 0.91, 0.84, 0.79, 0.64, and 0.76, respectively. After eliminating confounding factors, the both waist-to-hip ratio and baPWV were independent prognostic factors for ovulatory response to CC in patients with PCOS in multivariate analysis. Moreover, the measurement of waist-to-hip ratio and baPWV is quick and non-invasive to perform in comparison with blood sampling. Based on these results, both waist-to-hip ratio and baPWV might be candidates for prognostic factor for CC-resistant PCOS women.

In summary, the present study demonstrated that both metabolic and cardiovascular parameters were predictive for CC resistance in PCOS patients. The measurement of waist-to-hip ratio and baPWV may be useful tools to predict the possibility for ovulation in PCOS patients who receive CC. This study provides the first evidence of an association between arterial stiffness and CC resistance in patients with PCOS. A prospective study should be performed in order to access the clinical impact of measurement of baPWV for predicting CC-resistant PCOS women.

## Abbreviations

PCOS: Polycystic ovary syndrome; CC: Clomiphene citrate; baPWV: Brachial-to-ankle pulse wave velocity; hCG: Human chorionic gonadotropin; DHEA-S: Dehydroepiandrosterone sulfate; CV: Coefficient of variation; BMI: Body mass index; LDL: Low-density lipoprotein; HDL: High-density lipoprotein; FPG: Fasting plasma glucose; IRI: Immunoreactive insulin; OGTT: Oral glucose tolerance test; AUC: Area under the curve; HOMA-IR: Homeostasis model assessment of insulin resistance; QUICKI: Quantitative insulin-sensitivity check index; CVD: Cardiovascular disease; FAI: Free androgen index; AE-PCOS: Androgen Excess and Polycystic Ovary Syndrome.

## Competing interests

The authors declare that they have no competing interest.

## Authors’ contributions

TT contributed to study conception and design, acquisition, analysis and interpretation of data and drafting of the manuscript. HI, SH, MA, KM and AH contributed to acquisition and analysis of data. HK contributed to drafting of the manuscript and critical discussion. All the authors approved the manuscript.
